# Mucin-derived *O*-glycans supplemented to diet mitigate diverse microbiota perturbations

**DOI:** 10.1038/s41396-020-00798-6

**Published:** 2020-10-21

**Authors:** K. M. Pruss, A. Marcobal, A. M. Southwick, D. Dahan, S. A. Smits, J. A. Ferreyra, S. K. Higginbottom, E. D. Sonnenburg, P. C. Kashyap, B. Choudhury, L. Bode, J. L. Sonnenburg

**Affiliations:** 1 Department of Microbiology and Immunology, Stanford University School of Medicine, Stanford, CA USA; 2 Department of Gastroenterology and Hepatology, Mayo Clinic, Rochester, MN USA; 3 GlycoAnalytics Core, University of California, San Diego, CA USA; 4 Division of Neonatology and Division of Gastroenterology and Nutrition, Department of Pediatrics, University of California, San Diego, CA USA; 5 Chan Zuckerberg Biohub, San Francisco, CA 94158 USA

**Keywords:** Microbial ecology, Diseases

## Abstract

Microbiota-accessible carbohydrates (MACs) are powerful modulators of microbiota composition and function. These substrates are often derived from diet, such as complex polysaccharides from plants or human milk oligosaccharides (HMOs) during breastfeeding. Host-derived mucus glycans on gut-secreted mucin proteins serve as a continuous endogenous source of MACs for resident microbes; here we investigate the potential role of purified, orally administered mucus glycans in maintaining a healthy microbial community. In this study, we liberated and purified *O*-linked glycans from porcine gastric mucin and assessed their efficacy in shaping the recovery of a perturbed microbiota in a mouse model. We found that porcine mucin glycans (PMGs) and HMOs enrich for taxonomically similar resident microbes. We demonstrate that PMGs aid recovery of the microbiota after antibiotic treatment, suppress *Clostridium difficile* abundance, delay the onset of diet-induced obesity, and increase the relative abundance of resident *Akkermansia muciniphila*. In silico analysis revealed that genes associated with mucus utilization are abundant and diverse in prevalent gut commensals and rare in enteric pathogens, consistent with these glycan-degrading capabilities being selected for during host development and throughout the evolution of the host–microbe relationship. Importantly, we identify mucus glycans as a novel class of prebiotic compounds that can be used to mitigate perturbations to the microbiota and provide benefits to host physiology.

## Introduction

The luminal surface of the gastrointestinal tract is covered by a viscous mucus layer, which serves as the primary interface at which the host interacts with a dense microbial community. Secreted by host goblet cells, mucus is largely composed of highly glycosylated mucin proteins. The gel-like mesh of mucins provides both a barrier to shield the host from direct interaction with microbes, preventing inflammation [1], but also provides an energy-rich substrate for the microorganisms that reside in the gut [2, 3]. Degradation of the diverse chemical linkages within endogenous glycans requires a specialized set of glycoside hydrolases (GHs), reflected in the genetic composition of the gut microbiota. Notably, species prevalent in the human gut microbiota often possess broad glycan-degrading capabilities while “specialist” species may have narrower glycan-degrading potential [4].

The structural features of intestinal mucus glycans are strikingly similar to those of human milk oligosaccharides (HMOs) [5]. Mucin glycans are built upon an *N-*acetyl-galactosamine that is *O*-linked to serine and threonine residues of the mucin protein, while HMOs are built upon a lactose core structure universally present at the reducing end of these glycans [6]. In both mucin glycans and HMOs, the priming carbohydrate structure is extended with galactose-*N-*acetylglucosamine disaccharides and chains often terminate with fucose or sialic acid residues [7].

HMOs are the third most abundant compound in breast milk, after lactose and fat [8]. Colostrum is particularly rich in HMOs, the concentration ranging from 20 to 25 g/L in the first milk produced and decreasing to 5–20 g/L in more mature milk [9–11]. Numerous and diverse beneficial effects have been attributed to HMOs in breast milk during infant development [12–20].

Recently, extensive research has focused on the potential prebiotic role of HMOs. HMOs are indigestible by humans and are degraded throughout the gastrointestinal tract of breast-fed infants [21], becoming the primary microbiota-accessible carbohydrates available in the newborn diet. Species belonging to the genera *Bifidobacteria* and *Bacteroides* are optimal HMO-consumers: *Bifidobacterium longum* subsp. *infantis* (*B. infantis*) and *B. bifidum* encode in their genomes clusters of genes dedicated to HMO utilization [22–24]. *Bacteroides thetaiotaomicron* (*Bt*), described as a “generalist glycan-consumer” due to the numerous GHs encoded in its genome, grows efficiently on HMOs in vitro [5, 25]. In vivo studies with gnotobiotic mice revealed that lacto-*N*-neotetraose, one of the most abundant oligosaccharides in HMOs, provides an advantage to *B. infantis* over *Bt* in gut colonization [5]. However, the in vivo effect of the structurally diverse HMO pool in microbiota composition remains under-explored.

In the infant gut, MAC-consuming bacteria that reach the intestinal tract must rely primarily on HMOs or mucus as growth substrates. Interestingly, transcriptional data demonstrates that HMO utilization in *Bacteroides* and *Bifidobacterium* relies on pathways that also play a role in mucus utilization [5]. As HMOs have been described to confer numerous benefits to infants and similarities between microbial metabolism of HMOs and mucin glycans has been shown, we investigated the extent to which mucin glycans are able to confer benefits to the microbiota and host. We hypothesized that targeting convergent glycan-utilization pathways in the adult gut community with exogenously administered porcine mucin glycans (PMGs) could mitigate perturbations to the microbiota.

Empirical studies of the human microbiota can be performed in a controlled environment by colonizing germ-free (GF) mice with specific strains of bacteria (gnotobiotic) or a complete microbial community from human feces (humanized). The humanized mouse model recapitulates the vast majority of human microbiota compositional and functional features [26, 27]. In this work, we use both gnotobiotic and humanized mice to establish that the gut microbiota efficiently consumes HMOs. Furthermore, we demonstrate that a complex mix of glycans isolated from porcine mucin recreates some of the effect of HMOs on the gut microbiota and mitigates the negative effects of various community perturbations including antibiotic treatment, pathogen invasion, and a high-fat diet (HFD).

## Results

### HMOs are consumed by members of the microbiota, conferring a growth advantage to *Bifidobacterium* over *Bacteroides* in vivo

**Fig. 1:**
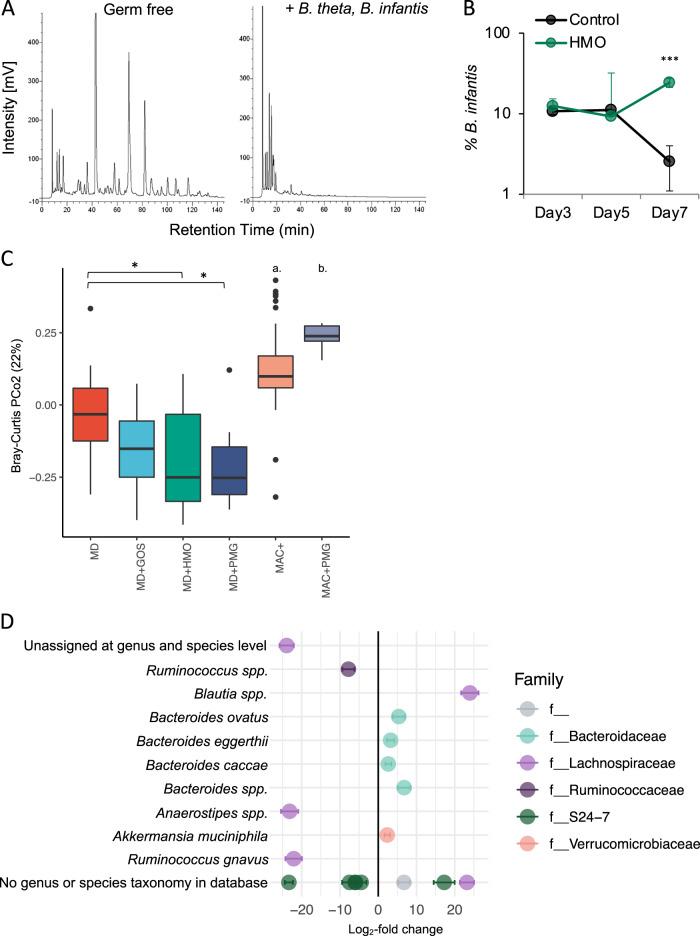
Human milk oligosaccharides (HMOs) are consumed by and shape the commensal microbiota. **a** Germ-free and mice bi-colonized with *Bt* and *B. infantis* were fed MD diet supplemented with 1% HMOs (w/v in water). HPLC-FL-based chromatograms of glycan content in stool samples at day 7 reveal degradation of HMOs in vivo. **b** HMO supplementation provides a competitive advantage to *B. infantis* over *Bt* in bi-colonized mice (*n* = 4 mice/group, mean ± SEM, ***P < 0.001, unpaired *t*-test). Abundance of *Bt* and *B. infantis* from feces was determined with CFU dilution plating. **c** Bray–Curtis dissimilarity metric indicates significant differences to community composition between mice on MD diet and MD diet supplemented with 1% PMGs, or HMOs. MD diet alone is not significantly different from MD diet supplemented with 1% GOS. (Data were combined across sampling timepoints: *n* = 12 MD, *n* = 19 MD + GOS, *n* = 21 MD + HMOs, *n* = 11 MD + PMG, *n* = 61 MAC^+^, *n* = 10 MAC^+^ + PMG. a. *MAC^+^ vs. MD; ****MAC^+^ vs. MD + GOS, MD + HMOs, and MD + PMG. b. ***MAC^+^ + PMG vs. MD; ****MAC^+^ + PMG vs. MD + GOS, MD + HMOs, and MD + PMG. ***P < 0.001, ****P < 0.0001, pairwise *t*-tests with Bonferroni multiple hypothesis correction). **d** Individual ASVs with significantly different abundance due to HMO supplementation (positive log_2_-fold change) versus MD diet alone (negative log_2_-fold change, mean ± SEM, adjusted *P* value < 0.01, parametric Wald test). Highest resolution taxonomic assignment indicated to the left.

We and others have previously reported the ability of both *Bacteroides* and *Bifidobacterium* to utilize select HMOs in vitro [5, 28]; as such, we wished to determine whether complex HMOs isolated from human donors favored *Bifidobacterium* or *Bacteroides* within the context of the gut environment. 6-week-old GF mice were bi-colonized with two HMO-utilizing taxa common to the infant gut: *Bt* and *B. infantis*. Mice were fed a MAC-deficient (MD) diet supplemented with HMOs (1% in water, chosen to approximate the mass of HMOs consumed by human newborns, adjusted for body weight, see Methods) for 1 week. Feces and cecal contents were collected and milk glycans were measured by high-performance liquid chromatography (HPLC) from all samples. In bi-colonized mice, no HMOs were detectable, whereas GF mice samples revealed a high concentration of milk glycans in both the fecal (Fig. 1a) and cecal samples (Fig. S1A, B), demonstrating that HMO-utilizing members of the commensal microbiota deplete HMOs within the host large intestine.

In vitro, *Bt* grows better on mucus glycans than *B. infantis*, whereas *B. infantis* grows better on HMOs than *Bt* [5]. After 1 week, HMO supplementation resulted in an expansion in the population of *B. infantis* relative to *Bt* compared to mice on regular water (24.3 ± 3.12% versus 2.5 ± 1.45% on day 7; *P* < 0.001, *n* = 4 mice) (Fig. 1b). These results confirm that HMOs provide a selective advantage to *B. infantis* over *Bt* in vivo. Therefore, although HMOs are utilized by generalist glycan degraders, they can provide a competitive advantage to microbes that specialize in HMO utilization.

### HMOs shape the composition of the gut microbiota

To address the extent to which HMOs shape a complex microbial community, we administered purified HMOs to ex-GF mice colonized for 6 weeks with a human microbiota (humanized). Compared to either a standard mouse diet rich in MACs (MAC^+^) diet or MD diet alone, purified HMOs engender a distinct microbial community (Fig. 1c). HMO supplementation to MD diet led to the significant enrichment of the known mucin glycan degraders *B. caccae* and *Akkermansia muciniphila* [25], as well as polysaccharide generalists *B. ovatus* and *B. eggerthii* (Fig. 1d).

Purified HMOs are structurally diverse, comprised of many molecules with different chemical properties [6]. An important question is whether glycans with slight structural differences within HMO mixtures can differentially impact the community. To investigate whether structural nuance in exogenous glycans led to changes in the composition of the gut microbiota, we employed two of the most abundant glycans found in HMOs and administered them in pure form to humanized mice. Lacto-*N-*tetraose (LNT, Galβ1-4GlcNAcβ1-3Galβ1-4Glc) and Lacto-*N*-neotetraose (LNnT, Galβ1-3GlcNAcβ1-3Galβ1-4Glc) differ only in the placement of a linkage between galactose and *N*-acetylglucosamine (Fig. S1C). GF mice were humanized and then switched to MD supplemented with LNT or LNnT (1% w/v in water) or maintained on MAC^+^ with plain water.

Bray–Curtis dissimilarity metric reveals clear separation of microbiota composition between mice consuming a MAC^+^ diet from MD supplemented with either of the two HMOs. Moderate but significant separation in composition was also observed between LNT versus LNnT supplementation in the MD diet (Fig. S1D). The switch from MAC^+^ to MD background diet drives high-level structural changes to the composition of the humanized gut microbiota (Fig. S1E). However, nuanced higher-resolution taxonomic changes occur corresponding to supplementation of one of two isomeric tetrasaccharides that differ in a single glycosidic linkage: nine taxa were significantly different at adjusted *P* value < 0.01 between LNT and LNnT supplementation (parametric Wald test [29], data not shown), including four ASVs belonging to the Bacteroidales family S24–7, *Coprococcus spp*. and *Sutterella spp*., two unidentified Erysipelotrichaceae (enriched with LNnT), and one unidentified *Blautia* strain (LNT). Accordingly, a Random Forests classifier predicts the three diets with 87.5% accuracy (out-of-bag estimate of error, with 30% class error for LNnT, 10% for LNT, and 0% for SD. Leave-one-out cross-validation results in accuracy of 94%), supporting the subtle but significant differences imparted onto the gut community by two isomeric oligosaccharides.

### Structural analysis reveals similarities between PMGs and HMOs

**Fig. 2:**
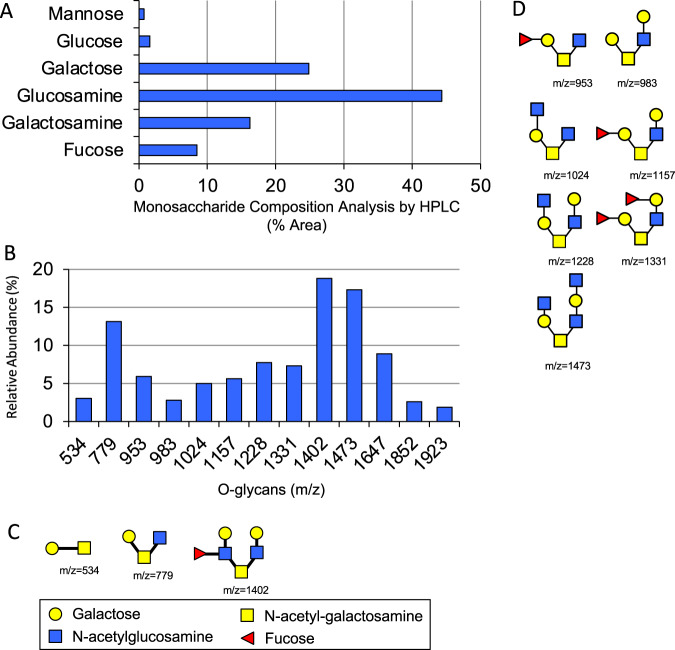
Structural analysis of porcine mucin glycans (PMGs). **a** Six detectable monosaccharides were identified after total acid hydrolysis of PMGs, with four monosaccharides associated with mucin glycans dominating. Amino sugars are the N-acetyl forms. **b** 13 abundant *O*-linked glycans were detected in purified PMGs using MALDI-TOF mass spectrometry. **c**, **d** Structures of the *O-*glycans quantified in (**b**) were predicted with GlycoWork Bench. Three of the identified PMGs (**c**) were validated with MS/MS.

HMOs are structurally similar to mucin glycans. We modified a described protocol [30] for purifying neutral *O*-glycans from commercially available porcine mucin using reductive ß-elimination followed by anion exchange chromatography, and characterized the purified material. Hydrolysis with trifluoroacetic acid (TFA) and HPAEC-PAD analysis revealed that PMGs are composed of units of glucosamine, galactosamine, galactose, glucose, fucose, and mannose, consistent with previous reports of mucin glycan composition ([31], Fig. 2a). The unexpected presence of mannose, a monosaccharide not commonly found in *O*-linked glycans is likely due to a small amount of liberated *N-*glycan during purification. MALDI-TOF mass spectrometry identified 13 major glycans (Fig. 2b), for which structures were predicted with Glycobench software (see Materials and Methods) based on m/z values, and three of thirteen were confirmed by MS/MS fragmentation pattern (Figs. 2c and S2). Structures were inferred for the remaining ten masses based on previous structural work on mucin glycans (Fig. 2d). Sialic acid content quantification from purified PMGs revealed an absence of *N*-acetylneuraminic acid and *N*-glycolylneuraminic acid (data not shown) consistent with anion chromatographic depletion of the negatively charged glycan fractions. Our analysis affirms the similarity between PMGs and HMOs both in terms of structure and constitutive components.

### PMGs and HMOs drive similar high-level microbial community changes

Given the structural similarities between PMGs and HMOs, we sought to determine how exogenously administered PMGs would affect the composition of the gut microbiota. We administered pools of PMGs (1% in water, to MD or MAC^+^ diets) or a common synthetic prebiotic, galacto-oligosaccharide (GOS, 1% in water to MD) and compared microbiota composition between these conditions and the HMO supplementation experiment described above. Similar high-level changes in the community were observed when HMOs, PMGs, or GOS were supplemented to a diet deficient in plant polysaccharides, indicating taxonomic changes at the family level are driven primarily by background diet (Fig. S3A). A switch from MAC^+^ background diet to MD led to a reduction in alpha diversity, which was not restored by the administration of any exogenous glycans tested (Fig. S3B). However, the administration of 1% PMGs to MAC^+^ background diet led to a significant increase in alpha diversity (Fig. S3B). To support this finding, we sought to determine whether a lower concentration of PMG supplementation would yield the same enrichment, and indeed found that 0.3% PMGs in water supplemented to a MAC^+^ diet led to a similar increase in alpha diversity (Fig. S3C).

While the first principal component of Bray–Curtis dissimilarity is driven by background diet (MAC^+^ versus MD), the second principal component reveals significant separation between communities supplemented with PMGs or HMOs from MD alone, whereas GOS supplementation is not significantly different from MD (Fig. 1c). The gap statistic predicts five clusters amongst the six diets (MAC^+^ ± 1% PMG; MD ± 1% PMG, HMO or GOS) (canonical correspondence analysis, method = firstSEmax [32]), providing further support for nuanced divergence of microbial communities in response to exogenous glycan administration. As seen with the administration of pure LNT and LNnT, administration of PMGs or HMOs to MD diet led to significant changes to the community at lower taxonomic levels. Both HMO (Fig. 1d) and PMG supplementation (Fig. S3D) led to the significant enrichment of the mucin-degrader *B. caccae* and an unidentified member of the *Blautia* genus. PMG supplementation to MD led to a significant increase in *B. eggerthii*, whereas *B. fragilis* and *B. ovatus* were enriched with PMG supplementation to MAC^+^ diet (Fig. S3E).

### PMGs accelerate recovery from antibiotic perturbation

**Fig. 3:**
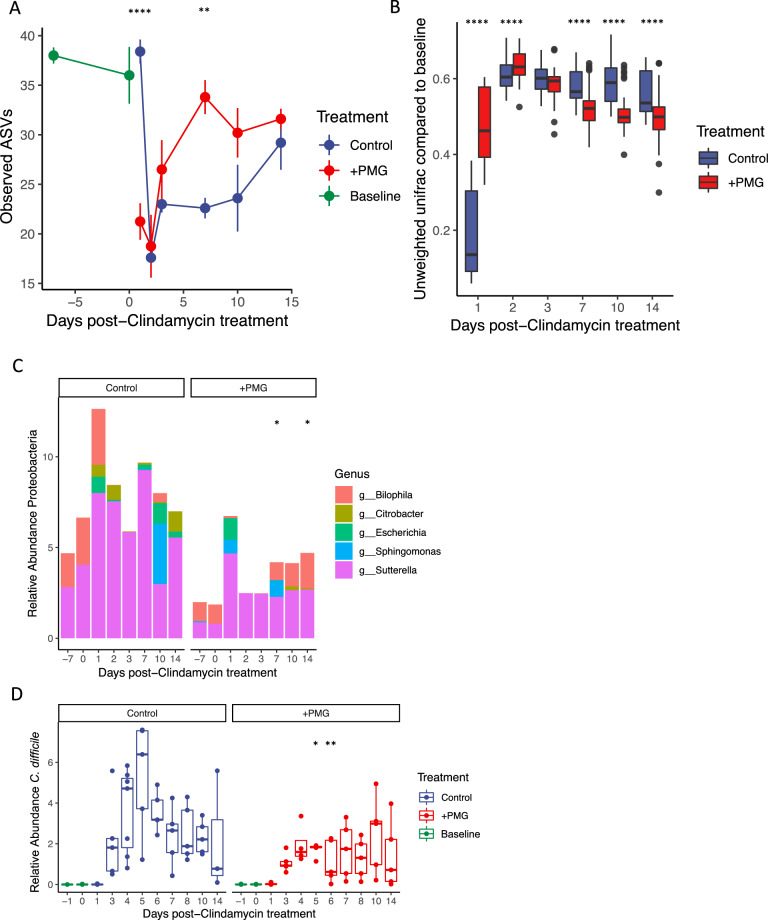
Treatment with PMGs leads to accelerated postantibiotic recovery. **a** MD diet supplemented with 1% PMGs (+PMG) leads to accelerated recovery of alpha diversity compared to MD diet alone (Control, mean ± SEM shown, **P < 0.01, ****P < 0.0001, pairwise *t*-tests with Bonferroni correction for postantibiotic treatment timepoints). Baseline alpha diversity measurements are during MAC^+^ diet prior to clindamycin treatment. **b** Unweighted UniFrac distance (compared to preantibiotic MAC^+^ baseline) reveals that PMG supplementation to MD diet (+PMG) leads to a faster trajectory back to baseline community than MD diet alone (Control, ****P < 0.0001, pairwise *t*-tests with Bonferroni correction). **c** Mean relative abundance (%) of the phylum Proteobacteria is suppressed in mice treated with 1% PMGs compared to the MD control. Bars are colored by the genus. Total Proteobacteria abundance is significantly higher days 7 and 14 postantibiotic treatment in the control group. (*P < 0.05, pairwise *t*-tests with Bonferroni correction). **d** Relative abundance of *Cd* is suppressed with PMG supplementation compared to MD diet alone (*P < 0.05, **P < 0.01, pairwise *t*-tests with Bonferroni correction).

We next pursued the investigation of purified PMGs to mitigate microbiota disturbance. Humanized mice were switched to MD diet and treated with 1 mg clindamycin concurrent with 1% PMG supplementation in water. When compared with no supplementation, PMG led to faster recovery of alpha diversity (Fig. 3a) and an accelerated trajectory to the baseline community microbiota as measured by comparison of UniFrac distance to preantibiotic timepoints (Fig. 3b). Furthermore, mice treated with PMGs exhibit a reduced bloom of Proteobacteria, a hallmark of postantibiotic oxygenation and inflammation in the gut (Figs. 3C, S4A [33, 34]). Additionally, PMG supplementation with antibiotics leads to a faster recovery of the relative abundance of *A. muciniphila* (Fig. S4B). These data suggest that exogenous glycans could be simultaneously administered with a course of antibiotics to aid in recovery to the microbiota.

### PMGs suppress *C. difficile* abundance

As PMGs augmented community recovery post antibiotics, we sought to determine whether exogenous PMGs would affect host susceptibility to an antibiotic-associated pathogen, *Clostridium difficile* (*Cd*). *Cd* depends on disturbance to the microbiota to cause disease [35, 36] and thus represented a suitable target to determine whether exogenous mucus glycans could protect the host from infection.

1% PMGs were supplemented to MAC^+^ or MD diet, concomitant with antibiotic treatment (1 mg clindamycin). Mice were gavaged 24 h later with 200 µL saturated overnight culture wild-type *Cd* 630 (day 1). Administration of PMGs to mice on a background diet devoid of complex polysaccharides significantly reduced the *Cd* burden as measured by 16S rRNA reads (Fig. 3d) and selective plating (Fig. S5A). PMGs supplemented to MAC^+^ diet did not affect *Cd* abundance (Fig. S5B). Exogenous PMGs in either background diet did not alter histopathological scoring (Fig. S5C, Table S1). Whether the inhibitory effect of PMGs towards *Cd* in a MD diet occurs via reshaping the microbial community in a way such that *Cd* suffers a competitive disadvantage, directly inhibiting *Cd* growth or toxin activity [37], or altering host immune signaling directly or indirectly via the microbiota remains to be determined.

### PMGs attenuate host weight gain due to HFD

**Fig. 4:**
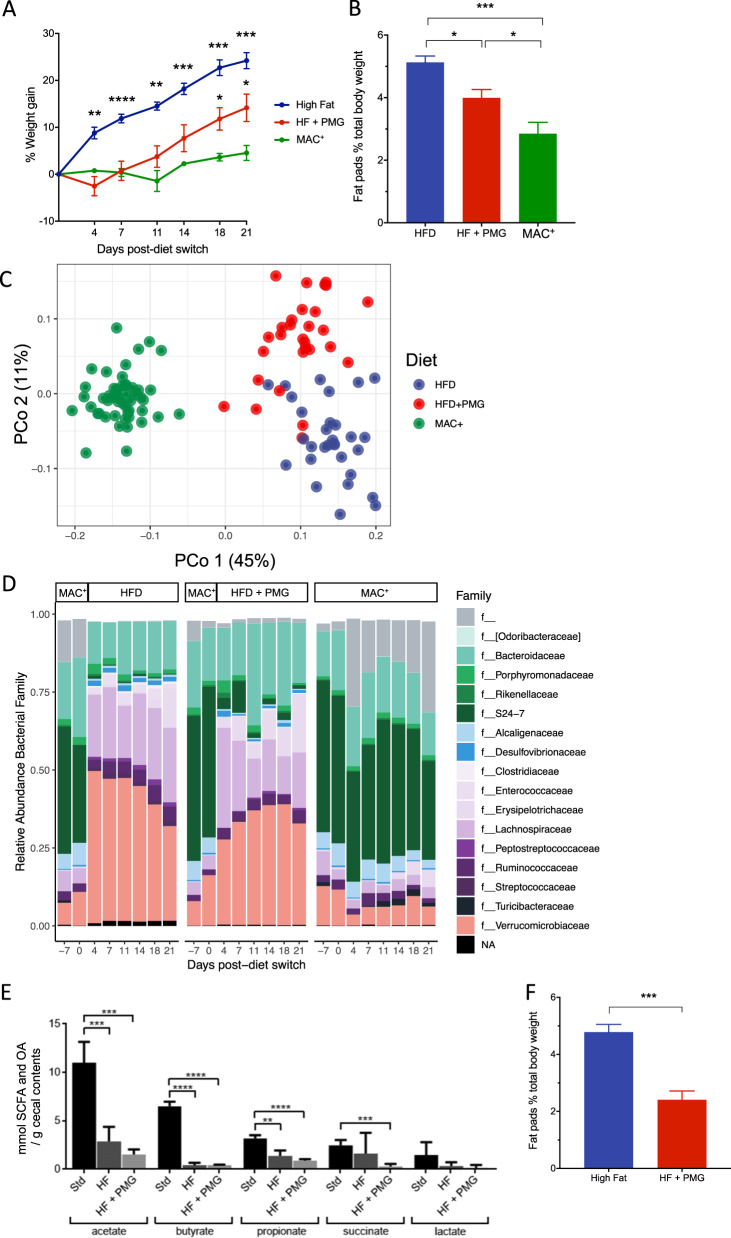
PMGs mitigate the effect of high-fat diet on host physiology and the gut microbiota. **a** Weight gain in individual mice fed HFD (*n* = 5 mice, blue), HFD supplemented continuously with 1% PMGs (*n* = 5, red), or maintained on MAC^+^ diet (*n* = 4, red). Weight was compared to baseline day 0 prior to diet switch (mean ± SEM, repeated measures two-way ANOVA with Dunnett’s multiple comparison’s test, asterisk indicates significance versus MAC^+^). **b** Fat pads as percent total body mass at day 21 (mean ± SEM, ANOVA). **c** Unweighted UniFrac reveals separation of the three diet groups; PMG supplementation to HFD leads to a unique microbial community from HFD alone. **d** Changes in the top 100 most abundant taxa over time for mice maintained on MAC^+^, HFD, or HFD supplemented with 1% PMGs. f__ indicates that a strain is not assigned at the family level in the Greengenes database; NA indicates lack of taxonomic assignment at the family level. **e** Diet-dependent decreases in cecal short-chain fatty acids and organic acids were determined by GC-MS in cecal contents of mice fed MAC^+^, HFD, or HFD + 1% PMG. **f** PMGs administered twice for 7-day durations in water (1% w/v) are sufficient to reduce host fat accumulation due to HFD. Fat pads as percent total body mass at day 35 (mean ± SEM, *n* = 5 mice/group). For **a**, **b**, **e**, **f**: *P < 0.05, **P < 0.01, ***P < 0.001, ****P < 0.0001.

As *A. muciniphila* has been associated with diet-induced obesity models previously [38–40] and appears to be manipulable with administration of PMGs, we were interested in whether administration of PMGs could attenuate the effects of an HFD on host physiology. Three groups of age- and sex-matched mice were fed either a standard diet, HFD (60% fat and 20% carbohydrates), or HFD supplemented with 1% PMGs continuously in drinking water and their weight monitored over 3 weeks. HFD induced significant weight gain compared to MAC^+^-diet fed controls, and PMG supplementation to HFD significantly reduced host weight gain (Fig. 4a) and development of adipose tissue (Fig. 4b), despite no differences between the groups in the amount of food consumed. No differences in glucose tolerance were observed between HFD-fed mice and PMG supplementation to HFD (data not shown).

Exogenous PMG administration to HFD led to a microbial community distinct from that of MAC^+^ or HFD alone (Fig. 4c). Although HFD induces similar family-level taxonomic changes with or without PMG supplementation (Fig. 4d), beta-diversity analysis reveals clear separation of the humanized microbial communities on the three diets, indicating strong diet-driven changes in community composition (Figs. 4c and S6A). Accordingly, Random Forests classifies microbiota samples into the three diets perfectly (HFD, HFD+PMG, MAC^+^), and regression on percent weight gain in individual mice from baseline perfectly predicts diet. Predictive features from the Random Forests models include many members of the family S24–7, genera *Ruminococcus* and *Eubacterium* (data not shown). Short-chain fatty acid (SCFA) and organic acid (OA) analysis demonstrates drastically reduced metabolic output of microbial communities on HFD with and without PMGs (Fig. 4e), indicating that the phenotypic effect of PMGs during HFD is not mediated by normalization of SCFA production.

To determine whether a shorter duration of PMG administration could attenuate host weight gain during HFD, humanized mice were switched to HFD and dosed with 1% PMGs for seven days at 1 and 4 weeks after diet change. Transient exogenous PMG administration led to significant reduction of fat accumulation (Fig. 4f) as well as diminished weight gain (Fig. S6B) compared to HFD alone, although biological variability in baseline weight gain prior to PMG supplementation may play a role in the extent of overall weight gain we observed. Two 1-week pulses of PMGs led to a distinct microbial community from HFD alone; gut microbiota composition of mice dosed with PMGs remains distinct from the HFD-only group even when PMGs are removed from water (Fig. S6C). Random Forests again perfectly classifies the microbial communities into the groups on HFD alone or HFD supplemented transiently with PMGs. HFD leads to dramatic changes to the relative abundance of bacterial families compared to MAC^+^ diet (Fig. S6D). Several taxa are significantly enriched due to exogenous PMG administration on HFD background, including *A. muciniphila* (Fig. S6E), *B. caccae*, and members of the Lachnospiraceae family, including *Dorea*, *Coprococcus*, *Coprobacillus*, *C. hathewayi*, *Blautia*, and *E. dolichum* (Wald test, adjusted *P* value < 0.0005, Fig. S6F), which were also many of the top predictive features of the Random Forests classification (Table S2). There are multiple potential mechanisms by which PMGs could lead to reduced weight gain in mice, which is an area of important follow-up investigation.

### Mucin glycan-utilization gene clusters are abundant in prevalent commensals and rare in pathogens

Given the structural similarities between PMGs and HMOs and the capabilities of PMGs to mitigate community disturbance, we hypothesized that mucus glycan utilization has played a role in host and commensal microbiota co-evolution. To explore this hypothesis, we performed a broad in silico search for candidate mucin-degrading carbohydrate gene clusters (CGCs) within the genomes of 4500 common human gut commensals in the HGM database [41]. A CGC was defined as the colocalization of at least two GHs that have been previously reported to act on mucin (Table S3), plus a transporter or transcription factor (TF) within the same genomic locus. The rationale for requiring two GHs per locus was to increase the stringency in identifying candidate mucin CGCs: while these GH families contain members that act on mucus glycan structures, some of these families may also contain members that do not target mucin glycans. Therefore, by requiring two candidate mucin-degrading GHs we sought to reduce the instances of false positives, accepting that some mucin-degrading CGCs containing only one of these GH families would be excluded.

**Fig. 5:**
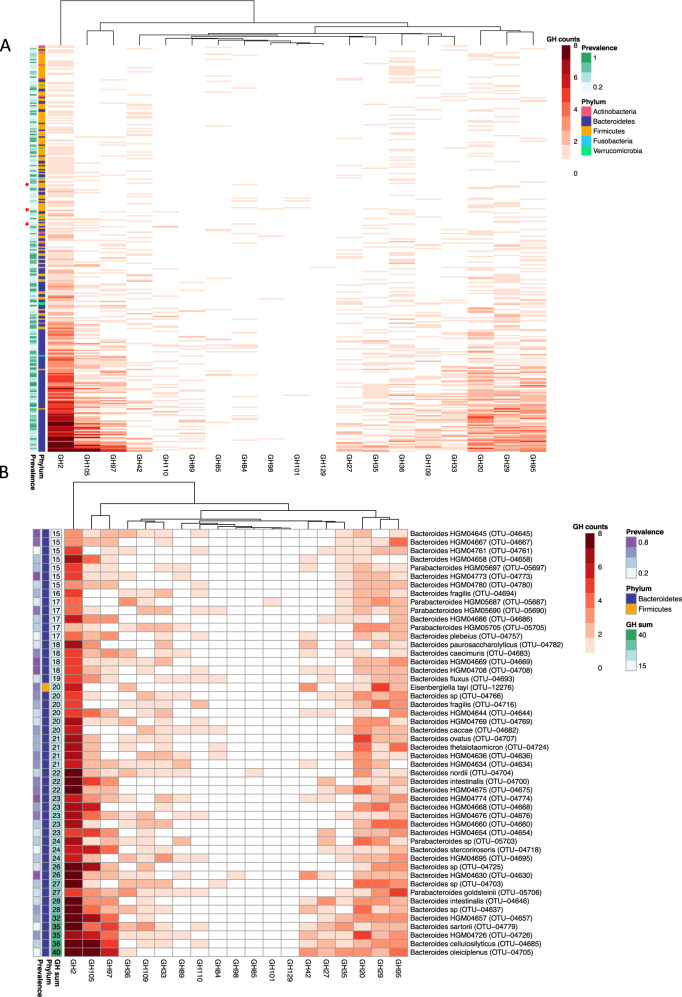
Prevalent gut commensals harbor high numbers of mucin-degrading carbohydrate gene clusters. **a** Numbers of putative mucin-degrading CGCs per genome in 839 taxa that met criteria for at least 1 mucin-degrading CGC and 10% prevalence in healthy humans. A healthy cohort from the Human Microbiome Project (HMP) dataset is used to define prevalence. Stars indicate the pathogenic strains queried that contained putative mucin CGCs. **b** The top 50 taxa within the HGM database harboring the highest number of mucin-degrading CGCs within their genomes. Total putative mucin-targeting glycoside hydrolases identified within these candidate mucin CGCs are indicated (GH sum).

The phyla Bacteroidetes and Firmicutes contain the taxa with the highest numbers of mucin-degrading CGCs per genome (Fig. S7). The genera *Bacteroides* and *Parabacteroides* contain the majority of strains with the highest number of mucin-degrading CGCs per genome (Fig. 5a). Many of the strains that were enriched in humanized mice with PMG supplementation (unidentified *Bacteroides* spp., *B. ovatus, B. caccae*) were identified as strains in the HGM with the highest numbers of candidate mucin-degrading CGCs (Fig. 5b). Proteobacteria, which were suppressed by PMG supplementation (Fig. 3c) harbor very few mucin-degrading CGCs (Fig. S7).

Prevalent human commensal bacteria show a greater diversity and abundance of mucus-degrading CGCs than well-known enteric pathogens. Of the 34 species within the HGM database that we identified as enteric pathogens (please see Materials and methods section and Table S4), only 4 met our criteria for harboring a candidate mucin CGC (*Vibrio vulnificus*, *C. botulinum*, *C. paraputrificum*, *C. perfringens*), as opposed to 835 gut commensals. Of these, 299 strains are also prevalent (>10%) in a healthy human cohort; only 3 are pathogens (Fig. 5a). Taken together these results show that mucus utilization is enriched in commensal, gut-associated bacteria and not common enteric pathogens, consistent with mucus as a key component of the symbiosis between host and microbiota within the gut.

## Discussion

During the first months of life, the delivery of glycans through breast milk is an influential force that shapes the gut microbiota of newborns and infants. It has been suggested that HMOs are involved in healthy brain and body development [12, 42], immune system maturation [13, 16], and protection against pathogens [14, 15, 17, 18]. Only small amounts of intact HMOs are found in the feces and urine of breast-fed infants [43], suggesting that most HMOs are hydrolyzed by neonate gut commensals [44]. Our experiments comparing gnotobiotic and GF mice support this hypothesis, and our results demonstrate that the gut microbial community indeed metabolizes HMOs. Furthermore, selective consumption of exogenous glycans can influence the composition of microbes present in the gut, favoring microorganisms with a high diversity of carbohydrate-active enzymes (e.g., *Bt* [5]) or with particular hydrolase activity to cleave certain glycan linkages (e.g., *B. infantis* [22]). The metabolic flexibility of polysaccharide generalists such as *Bt* may confer a competitive advantage during the transition from infancy to adulthood, as they may shift their metabolism from HMOs to dietary or mucin glycans [24].

The pool of HMOs found in breastfeeding mothers varies in composition, structure, and concentration day to day. When searching for molecules that aim to mimic the effect of HMOs on the gut microbiota, for example, in order to create an ideal infant feeding formula, similar structural complexity should be one of the major considerations. Our chromatographic mass-spectrometry analysis indicates that PMGs are a good candidate to mimic the complexity of HMOs. In vivo experiments with humanized mice indicate that PMGs and HMOs affect the microbiota in nuanced ways; the complex glycan mixtures structure the microbiota similarly at high-level ecological and taxonomic scales. A notably similar change due to supplementation with either glycan is an increase in the relative abundance of the species *A. muciniphila*, which relies on mucin as a carbon and nitrogen source, producing SCFAs from mucin fermentation [45]. Several studies in humans and animal models have documented the presence of *Akkermansia* with various positive phenotypes for the host. Studies in pregnant women have shown *A. muciniphila*-like bacteria to be positively correlated with normal weight gain over pregnancy [46] and reduction of diabetes [38]. In addition, *Akkermansia* levels have been inversely related to the severity of obesity [47] and inflammatory bowel disease [48]. Colonization of obese mice with *A. muciniphila* reduces body weight without dietary change and reverse diet-induced fasting hyperglycemia and insulin resistance index, reducing adipose tissue [40]. Notably, recent studies have associated *A. muciniphila* with positive responses to immunotherapy in cancer patients [49] and maintenance of intestinal adaptive immune responses during homeostasis [50]. Studies demonstrating an enrichment of Verrucomicrobia in industrialized compared to traditional populations [51] and blooming in mice fed a fiber-poor diet coincident with inflammatory markers [1, 25] suggests that context, quantification of absolute abundance, and perhaps function and strain-specific features of *Akkermansia* may be important considerations in overall health impact of this taxon within an individual.

The broad spectrum of glycan structures present in breast milk may represent an ecological strategy in which a robust gut ecosystem is selected for via maternal transmission of microbes supported by HMOs. By delivering a diverse pool of glycans, the mother facilitates establishment of a microbial community that may be more stable than one which relies primarily on limited structural diversity (i.e., a single type of glycan) or on fluctuating dietary input from the host. The HMO-consuming community can utilize structurally similar mucins; we show here that bacteria prevalent in healthy humans possess a diverse array of GHs capable of mucin glycan degradation. The manner by which the complex chemistries of exogenously-administered PMGs influence the microbial community in the background of endogenous human mucus glycans versus murine mucus glycans is an important area of follow-up investigation. Previous work has demonstrated detrimental effects of microbial degradation of the host mucus layer [1, 25]; exogenously provided mucus glycans may reduce the consumption of the host mucus in the absence of dietary MACs. The findings presented here support the idea that the host selects for a mucin-consuming microbial community to promote stability in the gut ecosystem. Accordingly, three diverse types of disturbance to the microbial community of humanized mice were mitigated by administration of exogenous PMGs. The close symbiotic interactions between commensals and their host appear to evolve at the mucus interface, with HMOs serving as important agents of this relationship after birth.

## Materials and methods

### HMO isolation from human milk

Human milk was obtained from 12 healthy volunteers recruited at the UCSD Medical Center, San Diego, CA, after approval by the University’s Institute Review Board. Proteins and lipids were removed from milk samples with centrifugation and ethanol precipitation. Roto-evaporation was used to rid samples of residual ethanol. Lactose and salts were removed by gel filtration chromatography over a BioRad P2 column (100 cm × 16 mm, Bio-Rad, Hercules, CA. USA) using a semi-automated fast protein liquid chromatography system.

### HMO isolation and purification from animal specimens

HMOs were extracted from mouse ileum and feces, purified over C18 and Carbograph microcolumns, fluorescently labeled with 2-aminobenzamide (2AB) and separated by HPLC on an amide-80 column (4.6 mm ID × 25 cm, 5 μm, Tosoh Bioscience, Tokyo) with a 50 mM ammonium formate/acetonitrile buffer system. The separation was monitored by a fluorescence detector at 360 nm excitation and 425 nm emission. Peak annotation was based on standard retention times and mass spectrometric (MS) analysis on a Thermo LCQ Duo Ion trap mass spectrometer equipped with a Nano-ESI-source.

### HMO profiling by high-performance liquid chromatography with fluorescence detection (HPLC-FL)

Isolated, dried HMOs from intestinal samples were fluorescently labeled with 2AB and cleaned with silica spin columns as previously described. The 2AB-glycans were separated by HPLC-FL on an amide-80 column (4.6 mm ID × 25 cm, 5 mm, Tosoh Bioscience, Tokyo) with a linear gradient of a 50 mM ammonium formate/acetonitrile buffer system. The separation was performed at 25 °C and monitored by a fluorescence detector at 360 nm excitation and 425 nm emission.

### Competitive colonization of gnotobiotic mouse

GF Swiss-Webster mice were reared in gnotobiotic isolators and fed an autoclaved MAC-deficient diet (BioServ, http://bio-serv.com) in accordance with A-PLAC, the Stanford IACUC. Mice were bi-colonized with overnight cultures of *Bt* and *B. infantis* using oral gavage as described in [5]. Subsequent community enumerations from mice were determined from freshly collected feces, by selective plating of serial dilutions on reinforced clostridial media (RCM) agar and brain–heart infusion blood agar supplemented with gentamicin (200 µg/mL).

### LNT and LNnT synthesis

Synthetic LNT and LNnT (Glycom A/S) were crystallized to a final purity of >99%. Characterization was performed using multiple methods including NMR (1D and 2D) mass spectrometry, and HPLC.

### PMGs preparation

PMGs were prepared as described by Martens et al. [30], with some modifications: *O*-glycans were released from porcine gastric mucin (Sigma Type III, 10% w/v) by incubation at 48 °C for 20 h in 150 mM NaOH with 750 mM NaBH_4_. The reaction was neutralized with HCl (10 M). Insoluble material was removed by centrifugation at 14,000 × *g* (30 min, 4 °C). Supernatant was filtered and dialyzed against dH_2_O with 1kD MWCO membranes (Spectra/Por 7, Spectrum Labs)) and subsequently lyophilized. Glycans were solubilized in 50 mM Tris pH 7.4 buffer and fractionated using DEAE-Sepharose CL-6B anion exchange columns.

### Monosaccharide and sialic acid determination from PMGs

For monosaccharide composition analysis, PMG samples were hydrolyzed using 2 M TFA at 100 °C for 4 h followed by removal of the acid under dry nitrogen flush. Dried samples were co-evaporated with 50 μL aqueous iso-propyl solution (50% IPA) twice to ensure complete removal of TFA. Finally, samples were dissolved in water and analyzed by HPAEC-PAD (Dionex ICS3000) using a CarboPac PA-1 (4 × 250 mm0 column with 100 mM NaOH and 250 mM NaOAc. Monosaccharides were identified with a sensitive postacceleration detector (PAD) using standard Quad potential as specified by the manufacturer.

Sialic acid content of PMGs was determined after hydrolysis with 2 M HOAc at 80 °C for 3 h. Acetic acid was removed by speed vacuum and free sialic acids collected by spin-filtration through a 3k MWCO filter followed by derivatization with 1,2-diamino-4,5-methyleneoxybenzene. Fluorescently labeled sialic acids were analyzed by reverse-phase HPLC using Acclaim120 C18 column (4 × 250 mm, 5 µ, Dionex) and a fluorescence detector with a slow gradient of 9–14% acetonitrile over 20 min.

### MALDI-mass spectrometry method for PMG structural analysis

Samples were prepared for MALDI-mass spectrometry with slight modifications to an established method [52]. To per-*O*-methylate, PMG samples were dissolved in dry DMSO, to which 100 µL sodium hydroxide in DMSO was added, followed by immediate addition of 200  μL methyl iodide. After continuous stirring for 1 h, a second aliquot of methyl iodide (50 μL) was added, followed by stirring for 30 min. The reaction was stopped with ice-cold water and the permethylated glycans were extracted with 1 mL chloroform. The chloroform layer was washed twice with water, dried, resuspended in methanol, mixed with sDHB matrix, and spotted on a MALDI plate. Spectra were acquired on positive mode, and putative glycan structures were assigned with Glyco Workbench [53, 54].

### Mouse humanization and enumeration

GF Swiss-Webster mice were humanized once with 200 µL of frozen human fecal sample resuspended 1:1 in anaerobic phosphate buffer saline (PBS) by oral gavage. The same adult human donor sample was used for all humanization experiments. The humanized microbiota was allowed to equilibrate for 4–6 weeks prior to onset of experimental manipulation. Mice were maintained in gnotobiotic isolators throughout the duration of the experiments and fed one of three diets: standard (Purina LabDiet 5K67), MAC-deficient (BioServ, http://bio-serv.com) or high fat (60% fat and 20% carbohydrates, D12492, Research Diets Inc.). When needed, 1 mg of clindamycin (Sigma) per mouse was administered via oral gavage or 200 µL of an overnight culture of *Cd* growth in RCM (BD Difco). To approximate the mass of HMOs available to breastfeeding infants, complex pools of glycans (purified HMOs and PMGs) were added at a concentration of 1% (w/v) in water unless otherwise specified. Infants consume ~7.5–11.25 g HMOs daily (~750 mL milk intake, HMOs are 10–15 mg/mL in breast milk), 1.5–2.25 g/kg (for a 5 kg infant). Mice consume about 5 mL water per day; at 10 mg/mL glycan concentration, this equates to 2 g/kg for a 25 g mouse. LNT and LNnT together constitute 15–20% total HMOs in breast milk [8]; as such, administration of pure glycans (LNT, LNnT) are provided at a higher concentration than at which they naturally occur. All animal protocols were in accordance with A-PLAC, the Stanford IACUC.

### *Cd* enumeration

For quantification of *Cd* CFU, 1 μL feces was serially diluted in PBS and plated onto selective media, composed of *Cd* agar base (OxoiD) with 7% v/v of defibrinated horse blood (Lampire Biological Laboratories), supplemented with 32 mg/L Moxalactam (Santa Cruz Biotechnology) and 12 mg/L Norfloxacin (Sigma-Aldrich) (CDMN). Plates were incubated overnight at 37 °C in an anaerobic chamber (Coy). Identification of colonies as *Cd* was validated by colony PCR, using the primers Cl1 (5′-TGTTGCAATATTGGATGCTTT) and Cl2 (5′-TGACCTCCAATCCAAACAAA), which target a fragment of *tcdB* gene.

### 16S rRNA amplicon sequencing and analysis

Fresh fecal samples were collected and frozen at −80 °C. DNA was extracted according to Earth Microbiome Project standard protocols using the Powersoil-htp extraction kit (MoBio). The 16S rRNA gene was amplified (515F, 806R) and sequenced using the Illumina MiSeq platform at the Medical Genome Facility, Mayo Clinic, Rochester, MN across 6 runs at 150 bp except the experiments with HFD, where 300 bp (Fig. 4a, c, d, S6A) and 250 bp (Figs. S6C–F) were generated. Raw reads were demultiplexed using QIIME 1.9 [55] and subsequently trimmed and denoised using DADA2 with standard input parameters maxN = 0, maxEE = 2, trunQ = 2 except for the GOS/HMO/PMG supplementation experiment (corresponding to Figs. 1c, d and S3) where maxEE = (5, 2) was used [56]. Taxonomy was assigned with the GreenGenes training set version 13.8 clustered at 97% identity. Phylogenetic trees were constructed by performing a multiple sequence alignment and constructing a generalized time-reversible with gamma rate variation maximum likelihood tree using a neighbor-joining tree as a starting point with the R packages *msa* and *phangorn* as described previously [57]. All resulting datasets were filtered for low-abundance ASVs at 10% prevalence and subsequently rarefied. R packages *phyloseq*, *DESeq2*, *RandomForests*, *rfUtilities*, *vegan*, *caret*, *cluster*, *harrietr, ggpubr, plotrix, rstatix*, and *ggplot2* were used for normalization, analyses, and visualization.

### Mining for mucin carbohydrate gene clusters in silico

Four thousand five hundred fifty-eight fully sequenced genomes were selected based on their identification as being human-associated (*n* = 4558, in the Human Gut MAG Species Database, HGMdb; https://github.com/snayfach/IGGdb) [41]. Carbohydrate-active enzyme assignment of GHs was performed using a Hidden Markov Model database [58, 59]. We identified physically linked CGCs using a default stringent parameter in the dbCaN software, which defines a CGC as physical linkage (distance ≤ 2) of at least two CAZymes with a TF or transporter; in this case, CAZymes were constrained to those listed in Table S3. GHs within CGCs were summed across each genome, and heat maps were generated using CGC counts. The prevalence of taxa across individuals was determined by running the IGGSearch software [41] with default settings on the 180 metagenomes available from healthy individuals in the HMP (https://hmpdacc.org/). Taxa were defined as pathogenic if they met the criteria of belonging to a list of genera (*Salmonella*, *Shigella*, *Yersinia*, *Vibrio*, *Campylobacter*) or species (*Cd, C. perfringens, C. botulinum, Pseudomonas aeruginosa*), and could be manually verified as pathogens based on existing literature (see Table S4 for a list of these 34 species).

### Body composition analysis

Animals were measured for total body fat mass and adipose deposits were precisely dissected and weighted. SCFA content was determined as described previously [60].

## Supplementary information

Supplemental figures and tables captions.

Table S1

Table S2

Table S3

Table S4

Figure S1

Figure S2

Figure S3

Figure S4

Figure S5

Figure S6

Figure S7

## Data Availability

Raw 16S sequencing data are available at the Stanford Digital Repository: https://purl.stanford.edu/kk642cf5780.

## Code availability

Code is available upon request.
