# Supplementary material for: Mucin-derived O-glycans supplemented to diet mitigate diverse microbiota perturbations
Source: ISME J. 2020 Oct 21;15(2):577–91. doi: 10.1038/s41396-020-00798-6 (PMC8027378; doi:10.1038/s41396-020-00798-6)
Supplement: Supplementary file 1 — Supplemental figures and tables captions. [file 41396_2020_798_MOESM1_ESM.docx]

**Figure S1. Corresponds to Figure 1. A)** HPLC-FL based chromatograms of glycan content in cecal contents of germ-free or bi-colonized mice fed 1% HMOs in drinking water. At day 7, mice bi-colonized with *Bt* and *B. infantis* degrade the HMOs still visibly present in germ-free mice. **B)** Glycan content from germ-free and bi-colonized control (*BtBi* = *B. theta*, *B. infantis*) mice not fed HMOs. **C-E)** Supplementation with two synthetic HMOs that differ in a single glycosidic linkage affects the composition of the gut microbiota. **C)** Structure of LNT and LNnT, two synthetic HMOs. Green circles indicate the differing location of the Gal-Glc linkage. **D)** Bray-Curtis distance reveals full separation of microbial communities of mice on MAC^+^ diet from MD supplemented with either synthetic HMO, as well as separation of LNT from LNnT (F_(2,29)_=41.736 * *P* < 0.05, **** *P* < 0.0001, one-way ANOVA with Tukey’s post-hoc comparisons). **E)** Relative abundance of bacterial families in MAC^+^ or MD supplemented with LNnT or LNT. f__ indicates that a strain is not assigned at the family level in the Greengenes database; NA indicates lack of taxonomic assignment at the family level.

**Figure S2. Corresponds to Figure 2.** Verification with MS/MS fragmentation pattern of the three porcine mucin glycan structures predicted by GlycoWork Bench from Fig 2C.

**Figure S3. Corresponds to Figure 1.** **A)**. Diet-induced changes to the relative abundance of bacterial families over time. All mice began on MAC^+^ baseline diet days prior to glycan supplementation. f__ indicates that a strain is not assigned at the family level in the Greengenes database; NA indicates lack of taxonomic assignment at the family level. **B)** There are no differences in two alpha diversity metrics between groups on MD dietary background; however, 1% PMG supplementation to MAC^+^ diet results in significant alpha diversity enrichment compared to MAC^+^ alone. Observed ASVs and Shannon index are higher in MAC^+^ +/- PMGs than MD supplemented with various glycans (observed ASVs: F_(5,128)_=17.382, *P* < 0.0001 one-way ANOVA with tukey’s post-hoc comparisons. a. * MAC^+^ vs. MD+GOS, **** MAC^+^ vs MD+HMOs, *** MAC^+^ vs. MD+PMG, *** MAC^+^ vs MAC^+^+PMG. Shannon diversity: F_(5,34.74)_=20.83, *P* < 0.0001 Welch’s ANOVA with Games-Howell post-hoc comparisons. b, **** MAC^+^+PMG vs. MD, MD+GOS, MD+HMOs, and MD+PMG.) **C)** More dilute PMG supplementation (0.3%) still leads to significant enrichment of alpha diversity to MAC^+^ diet (*** *P* < 0.001, Student’s t-test). **D)** 1% PMG supplementation (positive log_2_-fold change) to MD diet (negative log_2_-fold change) led to significant enrichment of several taxa that were also enriched by HMOs (mean +/- SEM, Wald Test, adjusted *P* value < 0.05). **E)** 0.3% PMG supplementation to MAC^+^ diet (as in S3C) led to enrichment of three *Bacteroides* species (mean +/- SEM, Wald Test, adjusted *P* value < 0.01).

**Figure S4. Corresponds to Figure 3. A)** Relative abundance of bacterial families after clindamycin treatment. f__ indicates that a strain is not assigned at the family level in the Greengenes database; NA indicates lack of taxonomic assignment at the family level. **B.** PMGs supplemented to MD diet lead to enhanced recovery of *A. muciniphila* (Pairwise t-tests with Bonferroni correction, * *P* < 0.05, *** *P* < 0.001).

**Figure S5. Corresponds to Figure 3.** **A)** Absolute abundance of *Cd* colonization enumerated with selective plating is reduced with PMG administration days 6 and 7 post-infection compared to unsupplemented control (MD diet, * *P* < 0.01, Student’s t-test). **B)** Relative abundance of *Cd* is not affected by PMGs in a MAC^+^ background. **C)** Blinded histopathological scoring of cecal (**i.**) and distal colon (**ii.**) tissues from mice infected with *Cd*.

**Figure S6. Corresponds to Figure 4.** **A)** PMG supplementation to HFD leads to distinct communities from HFD alone or MAC^+^ diet as quantified by the first principal component of unweighted UniFrac distance between communities (F_(2,107)_=782.603 **** P < 0.0001 ANOVA with Tukey’s post-hoc comparisons. n=30 HFD, n=29 HFD+PMG, n=51 MAC^+^). **B)** PMGs administered for 7-day durations in water (1% w/v) reduce host weight gain due to HFD. Weight gain compared to day 0 (mean +/- SEM, n = 5 mice/group, multiple t-tests). Red boxes denote period of two one-week pulses of PMGs administered to HFD + PMG group. **C)** Microbial communities of mice that were treated transiently with PMGs (red crosses) remain distinct from mice on HFD alone (blue), even when PMGs are removed from HFD (red circles). **D)** Relative abundance of the top 100 most abundant taxa on HFD alone or HFD with transient (1 week) PMG supplementation. Salmon-colored background boxes indicate sampling timepoints during which 1% PMGs were administered to the latter group. Both groups started on MAC^+^ diet (day 0). f__ indicates that a strain is not assigned at the family level in the Greengenes database; NA indicates lack of taxonomic assignment at the family level. **E)** *A. muciniphila* reaches a higher relative abundance in mice treated transiently with PMGs (** *P* < 0.01, t-test). **F)** Taxa that are significantly enriched due to transient PMG supplementation (positive) in water to HFD background (negative, mean +/- SEM, adjusted *P* value < 0.0005).

**Figure S7. Corresponds to Figure 5. A)** The distribution of number of putative mucin-degrading CGCs per genome amongst all phyla in the HGM database.

**Table S1.** Detailed results of blinded histopathological scoring for mice infected with *Cd*.

**Table S2**. Top 20 predictive taxa of the Random Forests classifier to predict HFD or HFD with transient PMG supplementation.

**Table S3.** List of literature references for mucin-targeting GHs[25, 61–64].

**Table S4**. List of bacteria categorized as pathogens for the mucin-glycan degradation analysis presented in Figure 5.
