# Supplementary figures and images for: Mucin-derived O-glycans supplemented to diet mitigate diverse microbiota perturbations
Source: ISME J. 2020 Oct 21;15(2):577–91. doi: 10.1038/s41396-020-00798-6 (PMC8027378; doi:10.1038/s41396-020-00798-6)

Figure S1. A

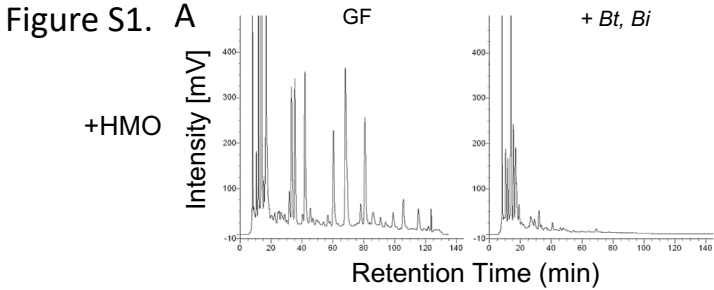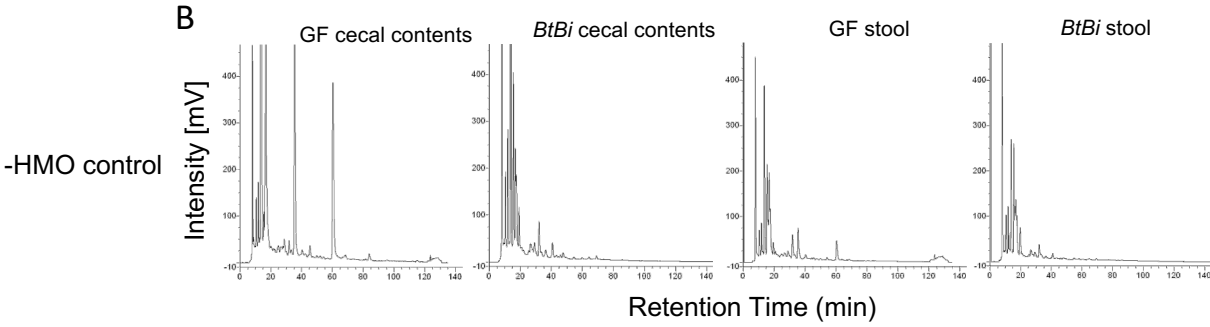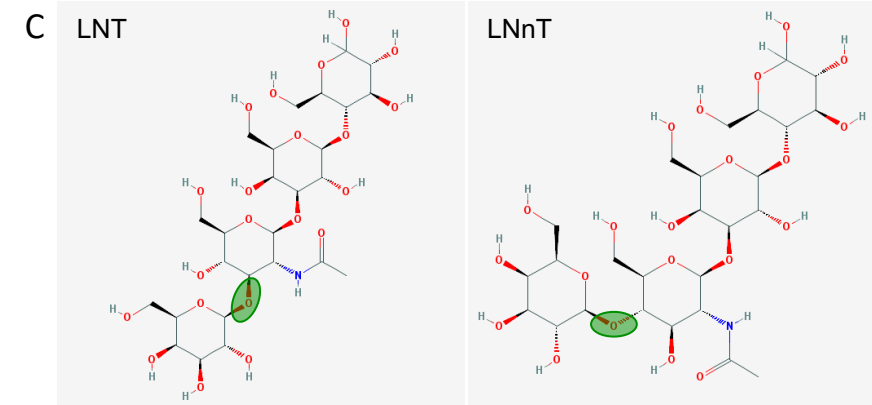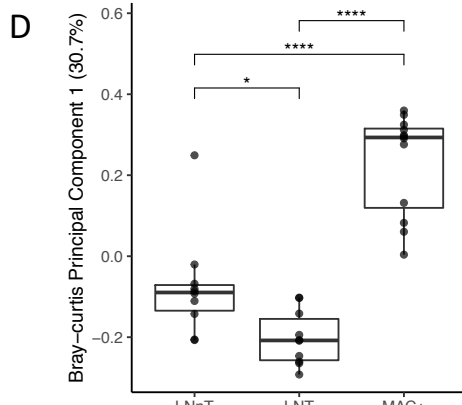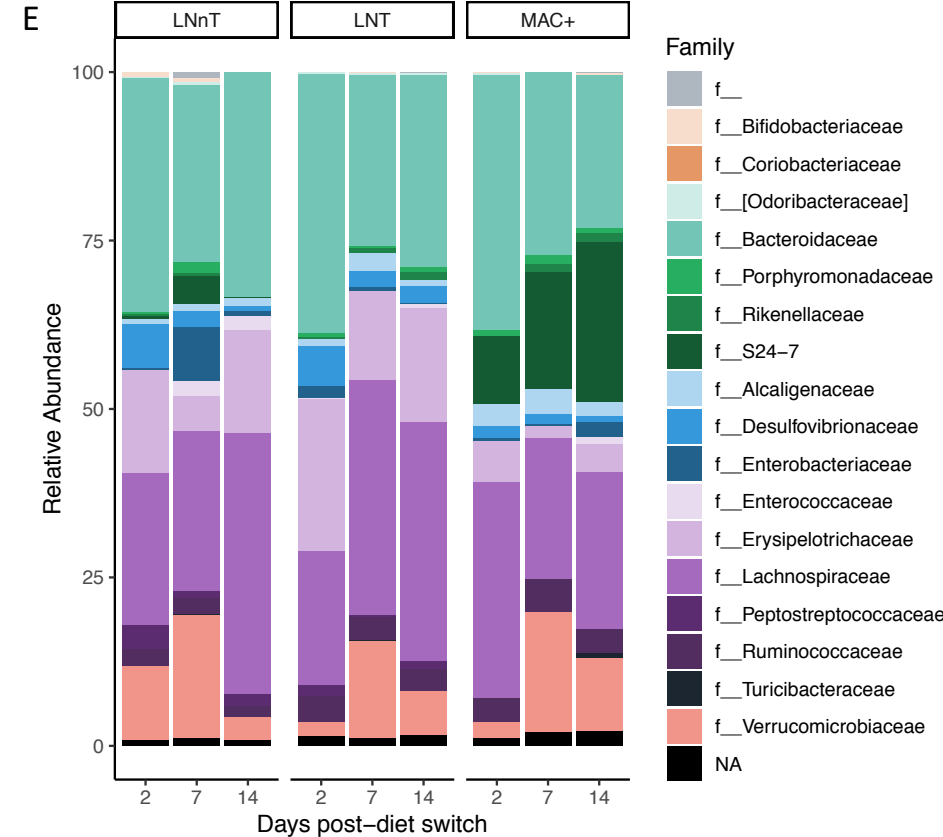

Supplement: Supplementary file 6 — Figure S1 [file 41396_2020_798_MOESM6_ESM.pdf]

Figure S2.

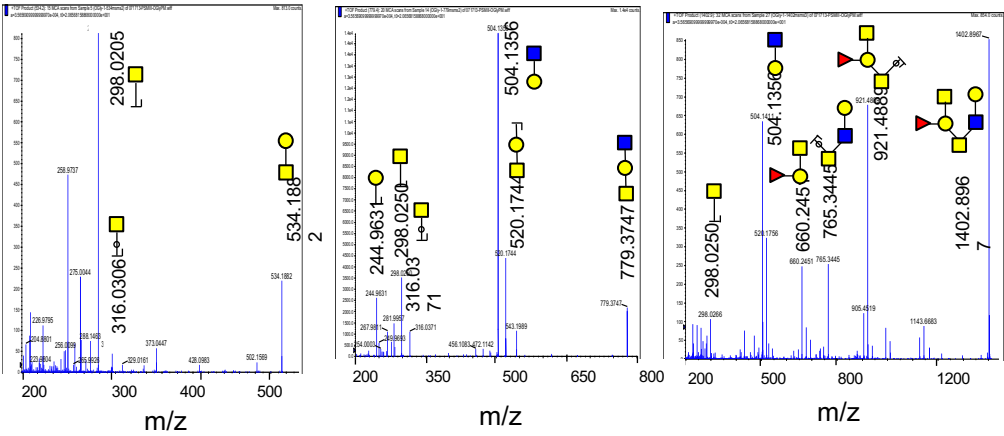

Supplement: Supplementary file 7 — Figure S2 [file 41396_2020_798_MOESM7_ESM.pdf]

Figure S3.

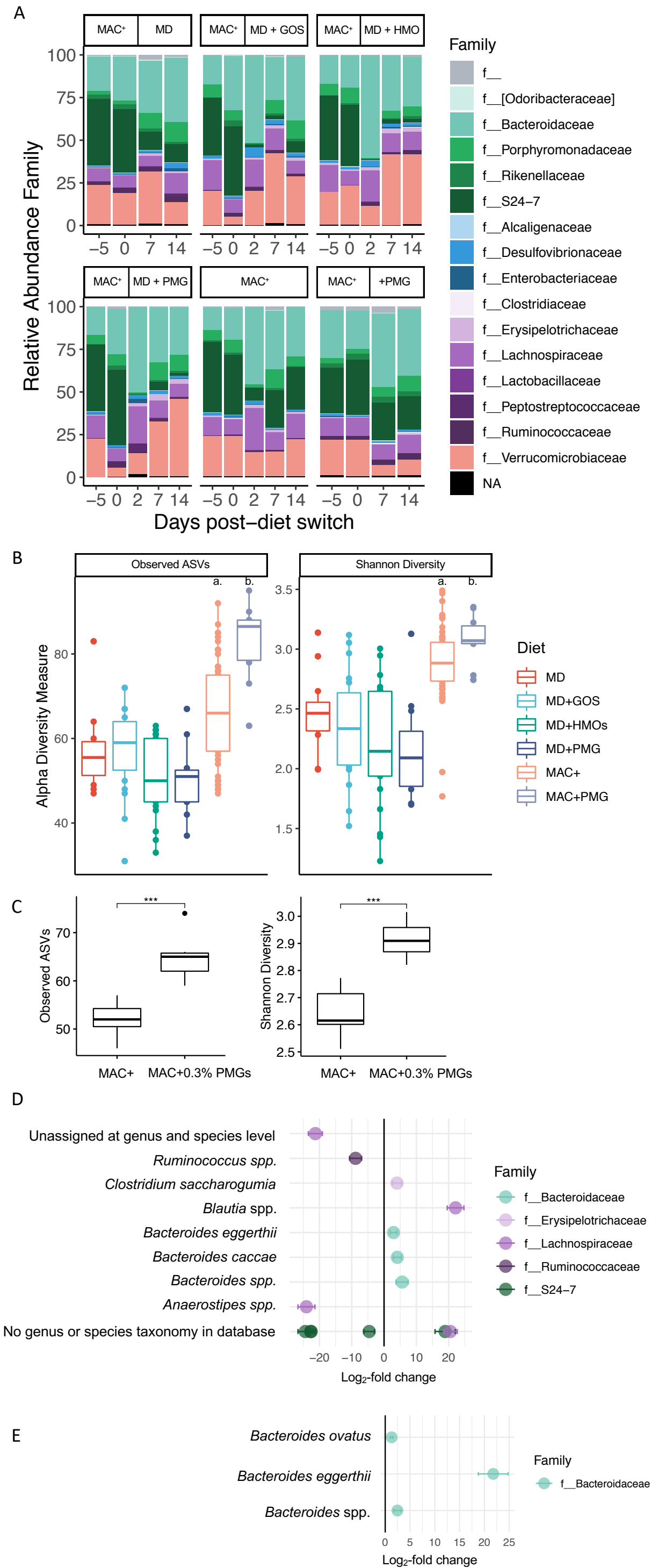

Supplement: Supplementary file 8 — Figure S3 [file 41396_2020_798_MOESM8_ESM.pdf]

Figure S4.

A

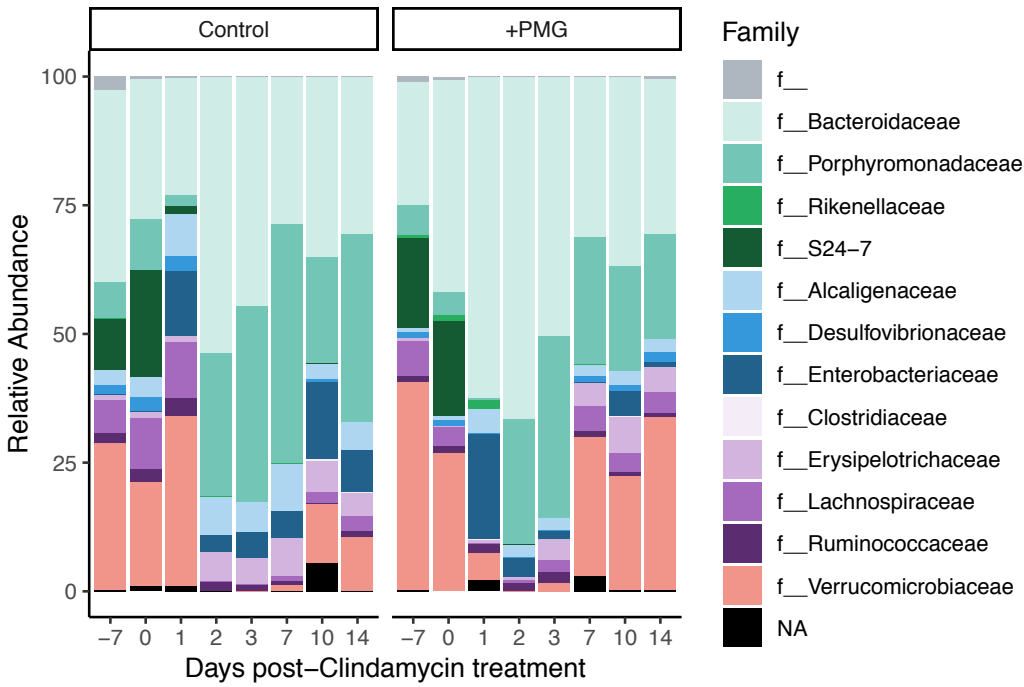

B

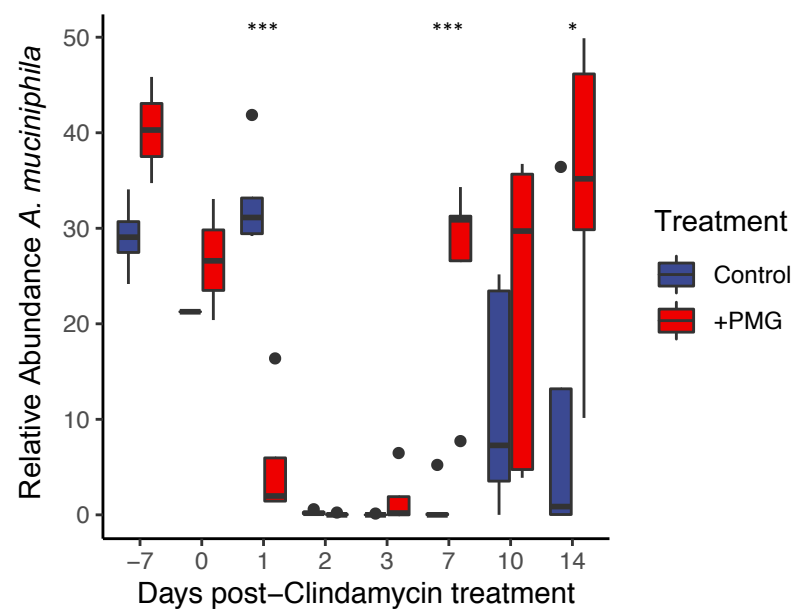

Supplement: Supplementary file 9 — Figure S4 [file 41396_2020_798_MOESM9_ESM.pdf]

Figure S5.

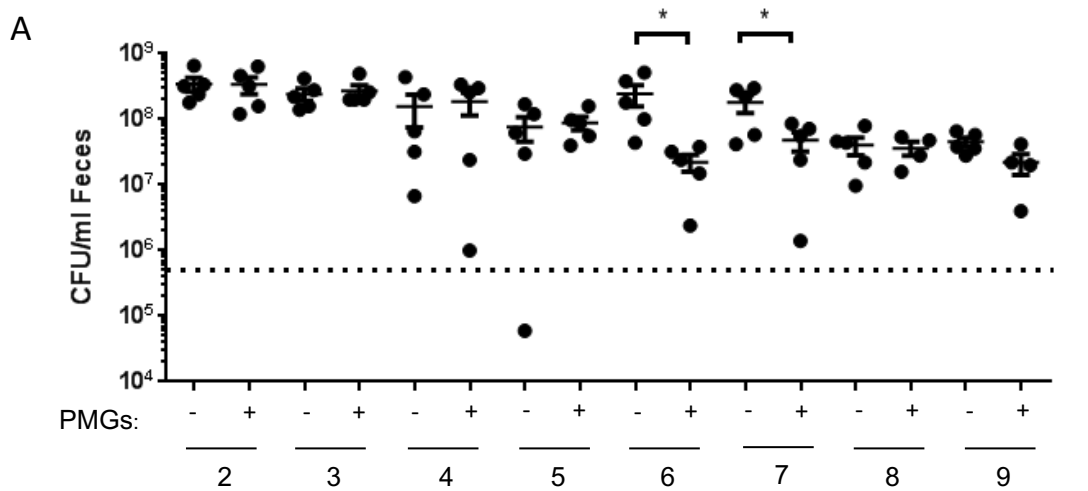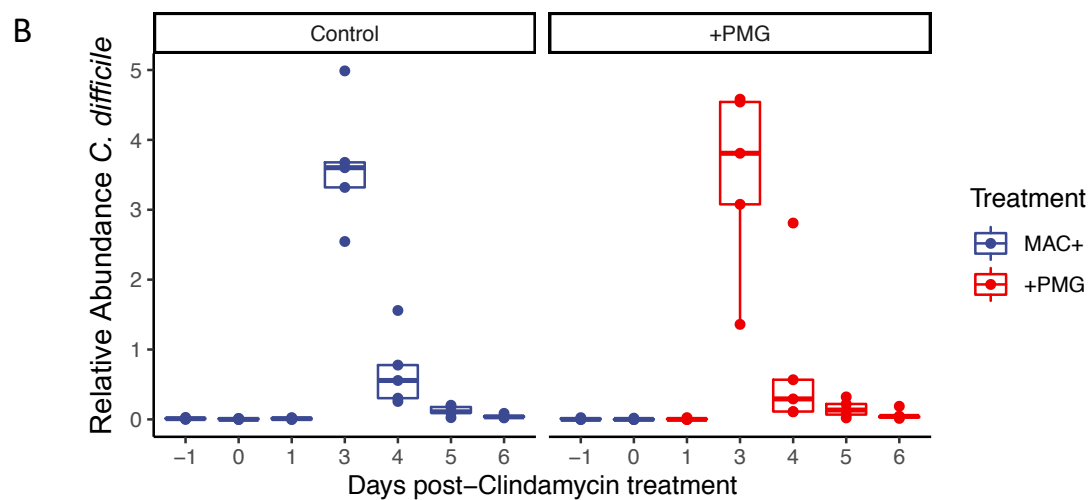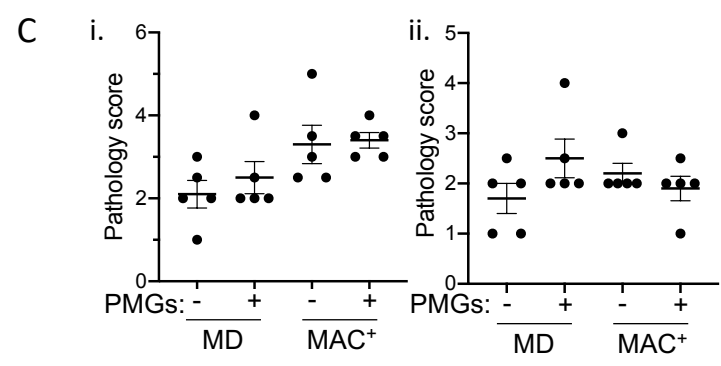

Supplement: Supplementary file 10 — Figure S5 [file 41396_2020_798_MOESM10_ESM.pdf]

Figure S6.

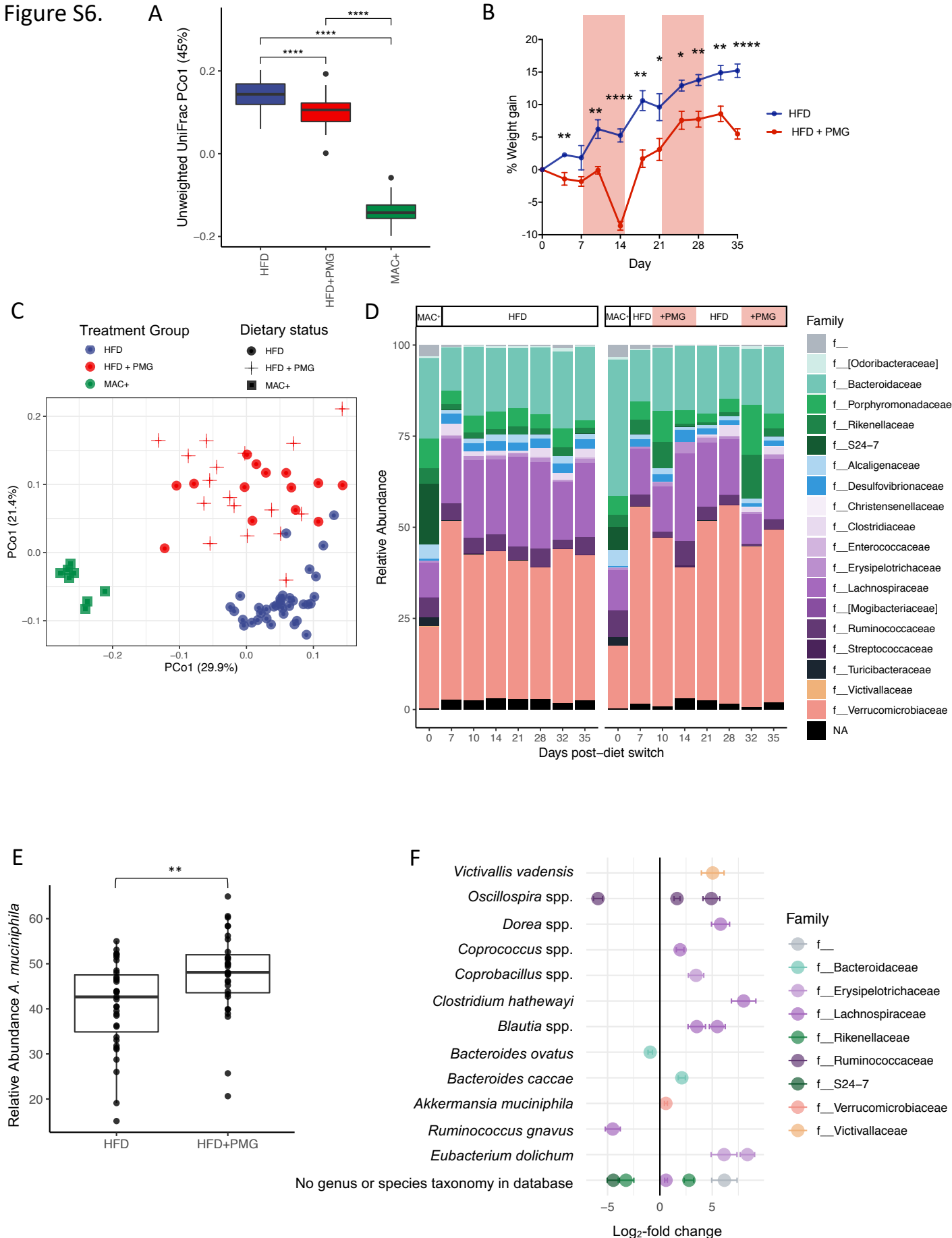

Supplement: Supplementary file 11 — Figure S6 [file 41396_2020_798_MOESM11_ESM.pdf]

Figure S7.

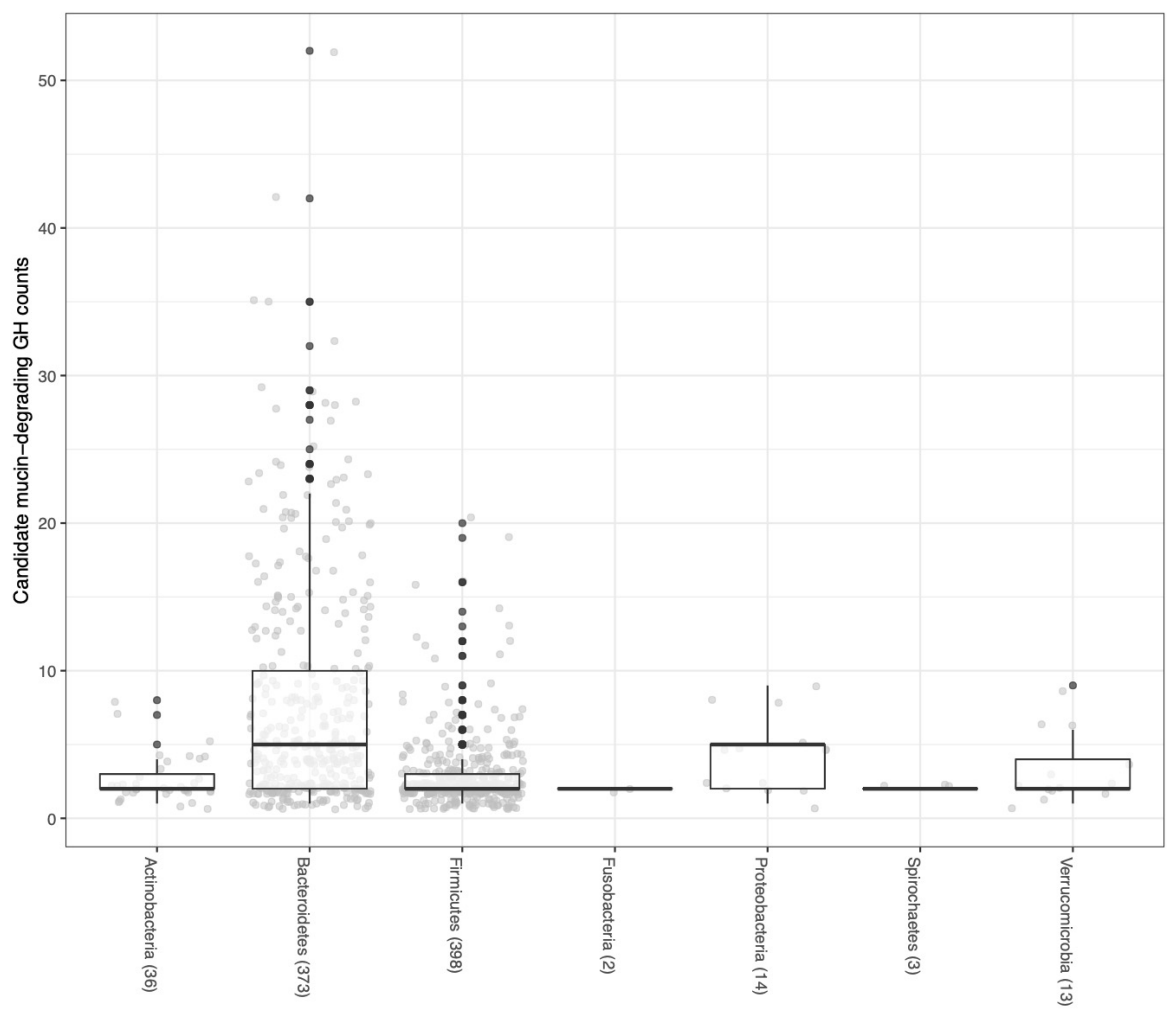

Supplement: Supplementary file 12 — Figure S7 [file 41396_2020_798_MOESM12_ESM.pdf]
